# Investigating Antiprotozoal Chemotherapies with Novel Proteomic Tools—Chances and Limitations: A Critical Review

**DOI:** 10.3390/ijms25136903

**Published:** 2024-06-24

**Authors:** Joachim Müller, Ghalia Boubaker, Norbert Müller, Anne-Christine Uldry, Sophie Braga-Lagache, Manfred Heller, Andrew Hemphill

**Affiliations:** 1 Institute of Parasitology, Vetsuisse Faculty, University of Bern, Länggass-Strasse 122, 3012 Bern, Switzerland; ghalia.boubaker@unibe.ch (G.B.); norbert.mueller@unibe.ch (N.M.); andrew.hemphill@unibe.ch (A.H.); 2 Proteomics and Mass Spectrometry Core Facility, Department for BioMedical Research (DBMR), University of Bern, Länggass-Strasse 122, 3012 Bern, Switzerland; anne-christine.uldry@unibe.ch (A.-C.U.); sophie.braga-lagache@unibe.ch (S.B.-L.); manfred.heller@dbmr.unibe.ch (M.H.)

**Keywords:** adaptation, mass spectrometry, model system, mode of action, resistance, target

## Abstract

Identification of drug targets and biochemical investigations on mechanisms of action are major issues in modern drug development. The present article is a critical review of the classical “one drug”—“one target” paradigm. In fact, novel methods for target deconvolution and for investigation of resistant strains based on protein mass spectrometry have shown that multiple gene products and adaptation mechanisms are involved in the responses of pathogens to xenobiotics rather than one single gene or gene product. Resistance to drugs may be linked to differential expression of other proteins than those interacting with the drug in protein binding studies and result in complex cell physiological adaptation. Consequently, the unraveling of mechanisms of action needs approaches beyond proteomics. This review is focused on protozoan pathogens. The conclusions can, however, be extended to chemotherapies against other pathogens or cancer.

## 1. Introduction

Modern therapeutic strategies require more than mere empirical evidence of documenting efficacy, namely insight into the macromolecular targets and the mechanisms of action related to these targets. In this respect, the fundamental paradigm “one compound—one target” issuing from simpler enzymological or receptor models still prevails [1,2]. This model predicts that an active compound interacts with one specific micromolar target, most likely a protein, thereby interfering with its biological function. Consequently, resistance to this compound should be linked to modifications of this target or to inactivation of the compound by protective mechanisms.

Evidence for the validity of this model seems to be legion, particularly in neurology and in infectiology in the case of viruses or bacteria. But, more recently acquired evidence has shown that this model is flawed. First, there is no therapy without side effects, meaning that secondary targets must be involved. Second, there are domains where the mechanisms of pathogenicity involve several interlinked biological processes, such as in cancer and infections with other eukaryotes—such as protozoan parasites—with a complex cellular organization similar to that of their hosts. Exposition to drugs may thus be countered by various strategies, including modifications of the cellular envelope, expression of transporters and detoxification mechanisms, deregulation of energy and intermediate metabolism, shift to a dormant state, and others. Prolonged exposition to drugs may result in drug adaptation or resistance, “fixing” successful strategies in an inheritable manner.

The rapid improvement of the methodology used to unravel the mechanisms of action of drugs has also caused doubts about the “one compound—one target” paradigm. During the last two decades, the analysis of genomes, transcriptomes, and proteomes has not only opened new doors to drug target discovery as anticipated in a seminal review article [3] but also made several quantum leaps with respect to sensitivity and the amount of data that can be handled within a decent time frame [4]. As we see it now, “the target” is part of a complex dataset rather than a defined “enzyme x” or “receptor y”.

Comparing proteomic investigations of antiprotozoal chemotherapies, we present two main strategies of target deconvolution, namely (i) the investigation of proteins binding to compounds of interest or “chemoproteomics” [5] and (ii) the determination of whole-cell proteomes of drug-resistant strains by shotgun mass spectrometry. We discuss the potential and the limitations of these strategies, concluding that they constitute only one element within a more holistic approach including physiological and morphological investigations. Based on our personal experience, this review will be focused on the diplomonad *Giardia lamblia*, a causative agent of persistent diarrhea [6], and on the apicomplexan parasite *Toxoplasma gondii*, responsible for abortions and neuronal disorders in humans and many animal species [7]. Both are zoonotic pathogens transmitted to humans mainly by ingestion of water or food contaminated by dormant stages: cysts in the case of *G. lamblia* [6,8] and oocysts or bradyzoites in the case of *T. gondii* [9]. Parallels to other species, in particular to *Neospora caninum*, which is closely related to *T. gondii* and responsible for abortions in cattle [10,11], and to *Plasmodium* sp., which is the causative agent of malaria, the most prominent disease caused by protozoans [12], will be drawn whenever helpful.

For this review, we have searched Pubmed (pubmed.ncbi.nlm.nih.gov) and Web of Science (webofscience.com) using the keywords adaptation, mass spectrometry, model system, mode of action, resistance, and target (accessed on 1 May 2024).

## 2. Proteomic Tools

### 2.1. General Remarks

The results of any empirical investigation are a function of the methodology employed for this investigation. With respect to investigations of biostructures, we all know that the term “higher resolution” indicates that more details can be visualized; thus, electron microscopy provides more structural information than light microscopy. In terms of proteins, “high resolution” means high sensitivity with respect to the correct identification of primary polypeptide sequences in solutions containing an increasing number of such polypeptides (i.e., low type II error) combined with high specificity (i.e., low type I error). As for all empirical tests, both errors cannot be reduced to zero. Consequently, false or non-reproducible results cannot be excluded [13].

### 2.2. From Protein Sequencing to Proteomics

In the case of enzymes, proteins can be identified and quantified by functional assays such as phosphatase, dehydrogenase, glucoside dehydrogenase, and others. Yet, “phosphatase activity” may occur due to a single protein species or to hundreds of different proteins. Consequently, the identification of proteins of interest is needed. The first method allowing the investigations of proteomes was thus the sequence determination of single, purified polypeptides (primary structure) by stepwise degradation from C- or N-terminal ends [14], facilitated by the development of liquid chromatography and one- and two-dimensional polyacrylamide gel electrophoresis post-1970 as highlighted elsewhere [15]. A large booster in terms of sensitivity and amount of generated data was the development of mass spectrometry (MS)-based methods. MS is the separation of charged particles in an electric field as a function of their mass-to-charge ratio (*m*/*z*). The first methodology applicable to a mixture of proteins—developed in the 1980s—is based on the indirect ionization of proteins embedded in a solid matrix via laser, the so-called matrix-assisted laser desorption ionization (MALDI). The energy transferred from laser via matrix on proteins ionizes them. The resulting ions are then separated according to their time of flight (TOF), lighter ions being faster than heavier ions, and their *m*/*z* is determined based on time used to arrive at the detector [16]. MALDI-TOF-MS is still used today, e.g., for the analysis of immunoprecipitated proteins [17], for protein profiling of food products [18], to identify causative agents of diseases such as bacteria [19], fungi [20], viruses [21,22], or for in situ proteomics in embedded tissues [23].

**Figure 1:**
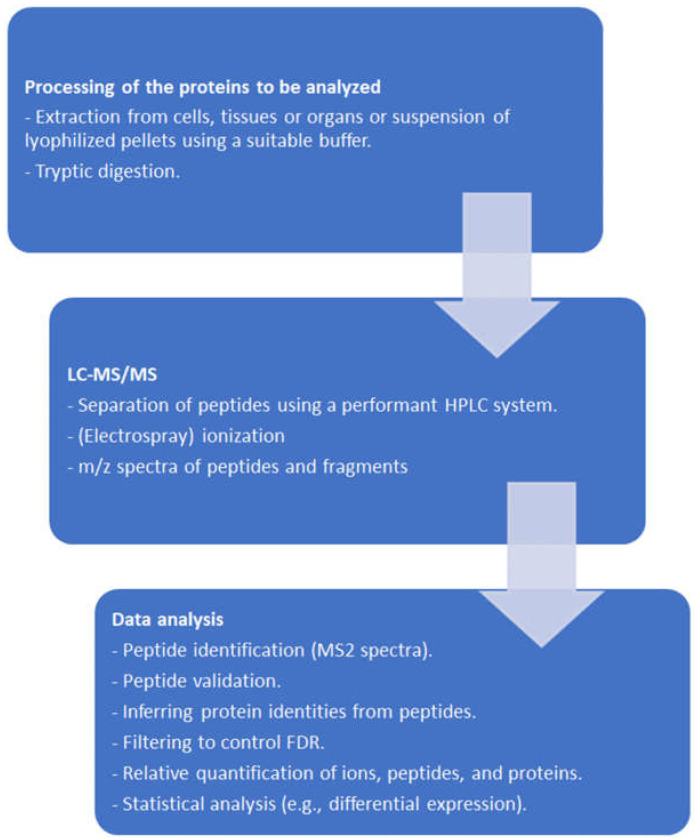
Simplified workflow of protein LC-MS/MS.

Peptides in liquid phase obtained after proteolytic digestion of a protein mixture, e.g., for whole-cell-shotgun analysis, are separated by liquid chromatography (“peptide fingerprinting” [24]), ionized by a convenient method, e.g., electrospray ionization (ESI), and injected into the MS. Within currently used MS, fragmented peptides undergo a second *m*/*z* determination. The procedure is therefore referred to as MS/MS. Instruments are constantly optimized with respect to resolution, scanning speed, sensitivity, and amount of data to be handled [25,26,27]. Ion mobility TOF, where ions are separated according to their collisional cross-section in the gas phase [28], is commercialized as timsTOF (Bruker, Bremen, Germany) and is one of the leading technologies to date (mid-2024). More detailed information can be found in specific reviews, such as, e.g., [29,30]. A simplified typical workflow is depicted in Figure 1.

Last but not least, it is noteworthy to mention recent achievements comprising proteomic analysis of single cells [31] and nanopore-based protein sequencing [32,33,34] even of single polypeptides [35]. Moreover, post-translational modifications can be investigated by LC-MS/MS. A good example is protein phosphorylation [36], as exemplified by studies on cancer cells treated with kinase inhibitors [37,38].

### 2.3. From Mass Spectra to Protein Data

As mentioned, the “raw” data of any MS-based analysis are the signal intensities as a function of the *m*/*z* ratios of the ionized peptides and/or their fragments. The identification and quantification of mass spectrometry data are routinely handled by complete software suites that return validated lists of quantified peptides and inferred proteins filtered to a false discovery rate (FDR) of 1%. Widely used examples of such software suites are Spectronaut (Biognosis, Schlieren, Switzerland; version 19), Fragpipe (most recent version 22) [39], and MaxQuant (v.2.6.2.0) [40]. A typical workflow consists of searching the MS2 spectra against theoretical spectra generated by digesting in silico protein sequences from a relevant protein database (e.g., Uniprot; www.uniprot.org; accessed on 1 May 2024). Candidate peptides of the correct mass are then scored by matching theoretical and experimental spectrum peaks, each software suite using different algorithms. The top scorer of each spectrum is retained as a potential match. Since even good matches may happen by chance, especially if the search space is large (large database, large number of post-translational modifications, etc.), many of these top scorers will be false identifications. Presently, the most widely used method to estimate the score distribution of false identifications is to concatenate the original target database with a set of decoy sequences, typically the reverse sequences of the original [41]. The decoys, which are always false identifications, are then used to model the false identification distributions of a number of measures such as scores, mass differences, retention time, spectral or chromatographic features, etc., which can be combined together to form a so-called discriminant score. An FDR can then be estimated per discriminant score threshold, and a selection of MS2 identifications filtered at 1% FDR returned. Note that due to the multiplicity of MS2 spectra mapping to the same peptide and peptide degeneracy (multiple proteins claiming the same peptide), the FDR must be re-calculated and controlled at all the levels (MS2 identification, peptide, protein) required by the experiment. Software suites will each use different procedures to return FDR-controlled lists of peptides and protein groups. While the latter will always be inferred from peptides based on Occam’s parsimony principle, its interpretation in practice can lead to potentially different protein group reporting [30].

Efforts are continuously made to boost the number of validated peptides and proteins by including further features in the estimation of FDR [42] and rescoring in specific application contexts [43]. Since correct proteins are often identified by multiple peptides and incorrect proteins by one random match only, a good practice is to require at least two unique peptides to consider protein X as “identified” [44].

Note that adding or removing samples from an experiment may alter the list of accepted peptides and proteins from a given sample since most tools will use the ion features and identifications of other samples to reinforce or weaken the probability of presence in a sample. Protein inference will also be evaluated differently based on the evidence from different sets of samples.

Actual quantification of the abundance of ions is performed in different manners depending on software and, crucially, on instrument acquisition mode and can be based on integrating MS1 peaks over time, counting corresponding MS2 spectra, or integrating corresponding fragment peaks. How to reconstitute protein intensities from constituent ions is usually considered at the same time as normalization, the latter being an essential condition of label-free quantification. A selection of some of the most frequently used measures of protein abundance are directly compared elsewhere [45]. For instance, the iBAQ (intensity-based absolute quantification) values are the sum of the peptide intensities divided by the number of theoretically observable tryptic peptides; iBAQ values have been shown to correlate with molar content and are normalized so that the intensity of one protein can be compared to that of another in the same sample. To compare the abundance of a given protein across different samples, normalization is usually done at the peptide level, under the assumption that most peptides have an unchanged abundance across samples. A popular measure of protein abundance, the Top3 value, sums up the normalized intensities of the three most abundant peptides of each protein. Considering for each protein only a limited number of peptides mitigates the problem that long proteins inevitably produce more peptides than short ones, which could bias the total intensity calculation. A widely used measure of protein intensity that is normalized for comparisons across samples is the LFQ (label-free quantification) value [39,40]. LFQ combines multiple peptide ratios and aims at stabilizing protein ratios between pairs of samples.

While normalization in label-free (LF) quantification aims at reducing various biases such as protein size, stability, varying total amounts between samples, and so on, more accurate quantification may be needed, in which case labeling approaches may be preferred. One common method established in the 1990s consists of feeding amino acids containing ^13^C instead of ^12^C or ^15^N instead of ^14^N to cell cultures (stable isotope labeling by amino acids in cell cultures, SILAC). The labeled proteins are extracted and mixed with unlabeled proteins of another culture. By analyzing the *m*/*z* shifts of labeled vs. unlabeled peptides, the amounts of the corresponding proteins in both cultures can be compared [46]. Another methodology is isobaric chemical labeling of soluble proteins or peptides using tags such as tandem mass tags (TMTs) or isobaric tags for relative and absolute quantification (ITRAQs) with defined *m*/*z* ratios upon ionization [47]. A direct comparison of LF with SILAC and TMT-based quantification reveals the best coverage for LF and the lowest performance for TMT [48].

The quantitative data may then be statistically analyzed using a suitable platform such as, e.g., Perseus [49]. To facilitate statistical analysis of protein quantities between samples, e.g., for differential expression studies, missing peptide values may be imputed. This can be done at peptide and/or at protein levels (see, e.g., [49]). Imputation procedures are based either on the assumption that a missing value is missing “not at random” (i.e., it has missed a detection threshold and can be assumed to be of small intensity) or “completely at random” (i.e., its original intensity can be low or high and its level of intensity is not known a priori). In the former case, the missing value is usually replaced by drawing a random number located near the lower left of the sample intensity distribution. In the latter case, the missing value could be replaced by calculating the maximum likelihood value from all other values. Imputing has the advantage of simulating natural variance and delivering log fold changes with a statistical measure even when a peptide or protein is absent from one condition. This approach may, however, be a source of errors, e.g., when missing peptides are imputed in knock-out compared to wildtype strains, or when strains expressing or not a transgene are compared (see, e.g., [50]). A good practice for setting significance criteria imposes a minimum log2 fold change of ≥1, a low value of the type I error after adjustment for the multiplicity, and, in general, lower than 0.05. Repeating the imputation several times and recording significance is a means to mitigate the stochasticity introduced by it, as detailed elsewhere [51]. Where two different normalized parameters are used for quantification of differentially expressed proteins (e.g., LFQ and Top3), belief in the data can be enhanced by considering only proteins with significantly different levels by both parameters. In any case, both the identification and the relative quantification of proteins by LC-MS/MS are probabilistic.

## 3. Affinity-Based Target Deconvolution

### 3.1. Functional and Binding Assays Using Isolated Proteins

Certainly, the most straightforward strategy to investigate the interactions between anti-infective compounds and their targets is based on functional or binding assays in the presence of isolated—in most cases recombinant—enzymes or receptors. In vitro functional assays allow identifying and optimizing ligands. This strategy has had some success in human vascular diseases [52], and high-throughput screening systems are available [53]. Another popular example of this strategy is the fight against HIV, where high-throughput screenings for inhibitors of essential steps of viral entry and proliferation are ongoing [54,55]. Well-known examples are the mandatory screening of drug candidates for interactions with the human ether-a-go-go K+ channel 1 (hERG1) in order to reduce the risk of potential side effects [56,57] or screening for compounds binding to acetylcholine receptors [58].

**Figure 2:**
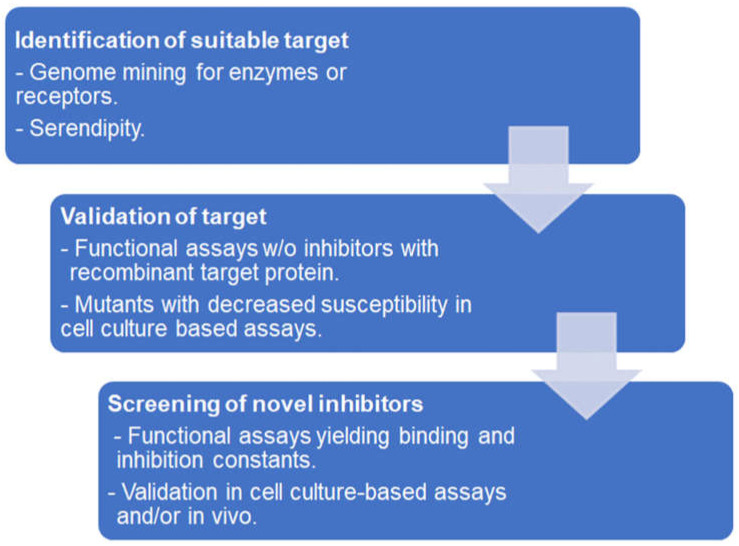
Workflow of antiprotozoal compound screening based on functional assays with recombinant proteins.

In the case of antiprotozoal compounds, the proteins of interest should be essential for the pathogen and irrelevant for the host. The first antiprotozoal compounds were discovered by trial-and-error testing of the available pharmacopeia or just by serendipity. A good example is the discovery of artemisinin antimalarials [59,60]. Modern target-based antiprotozoal drug development is based on the identification of a suitable target essential for the pathogen but absent from the host by genome mining, often combined with in silico modeling. Recombinant target proteins are produced in a suitable system, and functional assays are performed in order to determine and optimize the binding and inhibition constants of drug candidates. Of course, this method can also be applied to targets of existing drugs empirically identified, such as, for instance, anti-folates in the case of toxoplasmosis [61]. Ideally, the target is validated by overexpression and/or knock-out studies in a suitable model. Figure 2 represents this workflow.

If functional assays cannot be performed, ligand binding may be investigated using temperature shift assays. In fact, proteins with bound ligands are more resistant to chemical or thermic denaturation. Consequently, they remain in solution/soluble under conditions where proteins without ligands aggregate and can be precipitated by centrifugation [62]. Receptor and transporter antagonists are screened in whole-cell systems (e.g., human embryonic kidney cells 293T) expressing the respective recombinant target proteins [63,64]. Table 1 gives an overview of selected recent studies of inhibitor screenings in protozoal parasites.

As illustrated by this table, enzymes are elegant tools to identify suitable inhibitors via medium- to high-throughput screenings. This strategy has, however, its pitfalls, particularly if the target has been identified by mere genome mining. An example of such a pitfall is a phosphodiesterase (PDE) homolog identified in the genome of *Giardia lamblia* [79]. The recombinant catalytic domain of this homolog expressed in yeast is inhibited by a series of inhibitors, some of which also inhibit the proliferation of *Giardia* trophozoites in culture, suggesting that this enzyme may be a potential drug target against giardiasis [80]. The detail overlooked by the authors is that the protein encoded by the corresponding open reading frame is not present in trophozoites cultured under the same conditions as in the drug screenings (see the supplementary data in [81]). The fact that only PDE inhibitors with nitro groups inhibit proliferation [82] further reduces belief in PDE as a potential anti-giardial drug target. In fact, organic compounds containing nitro groups such as metronidazole or nitazoxanide commonly inhibit *Giardia* and other anaerobic organisms [83].

### 3.2. Affinity Chromatography

The main disadvantage of the strategy described above is that it is biased by the hypothesis concerning the mode of action and, thereby, the target of a class of compounds of interest. Therefore, in order to identify unknown targets, unbiased strategies are required. These chemoproteomic strategies exploit the binding affinities of proteins within the entire proteome of the pathogen as well as of the host in cases where side effects are investigated [84]. The oldest and best-known method is certainly affinity chromatography on a low- or medium-pressure liquid chromatography system. For this approach, the compound of interest is coupled onto an agarose or sepharose (trademark by Pharmacia, now Cytiva, Marlborough, MA, USA) column matrix via active groups such as epoxy, cyanogen bromide [85], carbomethyl, N-hydroxy-succinimide, [86] or others attached via a spacer [87], thus allowing free access by binding proteins. Cell-free crude extracts are then passed through the column. After abundant washes, binding proteins are eluted by ligands in solution via pH shift or increasing ionic strength. The eluted proteins are polished, e.g., by gel electrophoresis followed by subsequent sequencing of the most prominent bands, or can be directly identified by LC-MS/MS. A classic example of drug-binding proteins of pathogens identified by affinity chromatography and confirmed via binding and mutant studies are penicillin-binding proteins in *Escherichia coli* [88,89,90]. To enhance belief in the identified binding proteins as potential targets, controls minimizing false-positive results due to unspecific binding are paramount. This is valid for each methodology presented here.

Given the socio-economic importance of malaria, it is not surprising that a large number of chemoproteomic studies have been performed with a mind to identify targets of either well-established or novel antimalarials, as reviewed elsewhere [84]. A selection of studies based on affinity chromatography is listed in Table 2.

**Figure 3:**
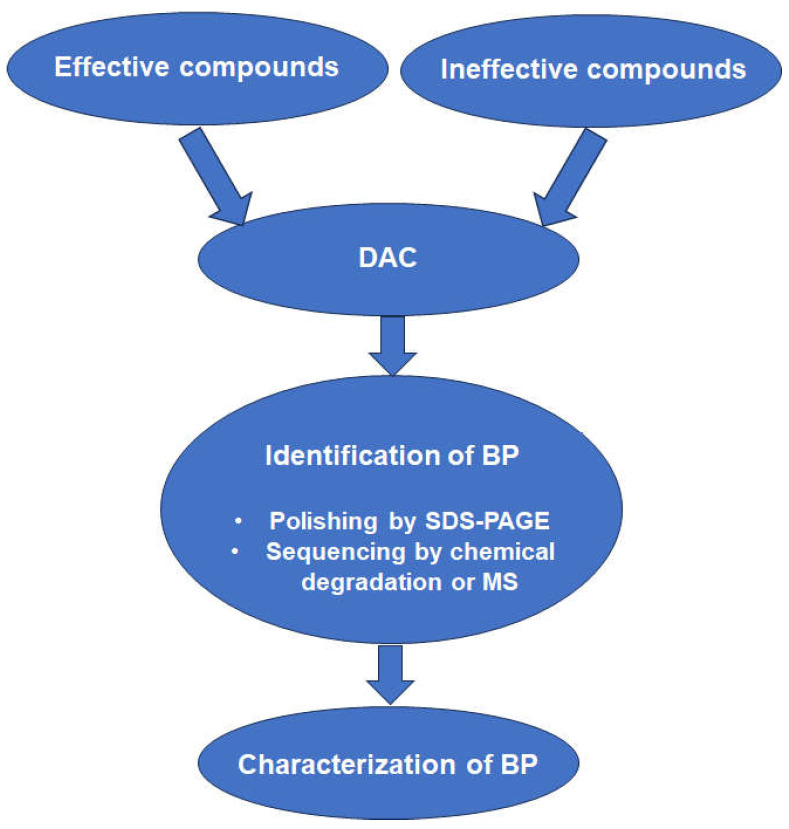
Workflow of differential affinity chromatography (DAC) studies listed in Table 2. For abbreviations, see Table 2. A more detailed scheme is given elsewhere [61].

A schematic representation of the workflow of the studies listed in Table 2 is given in Figure 3.

**Figure 4:**
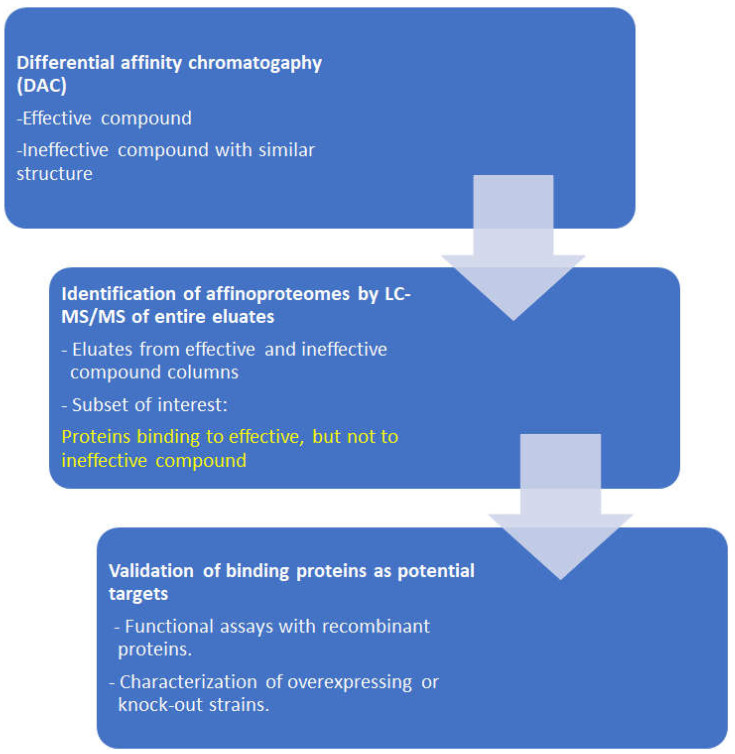
Workflow of differential affinity chromatography (DAC) studies as performed in Table 3.

During the last two decades, we have performed several target deconvolution studies in protozoal pathogens. In order to minimize the number of unspecific binding proteins identified due to the increased sensitivity of proteomic techniques, we perform differential affinity chromatography (DAC), where identical cell-free extracts are passed through a mock-coated column followed by a column coated either with the effective compound or with a ligand with high structural similarity but ineffective against the pathogen [61]. The workflow of DAC is illustrated in Figure 4.

The datasets of the studies obtained with entire eluates are much larger than those based on bands or spots excised from polyacrylamide gels (see also Table 3). This suggests that the concept of “one drug—one target” may be too simplistic or at least very optimistic. The fact that—where analyzed—a good number of host cell proteins are identified within the affinoproteomes of antiparasitic drugs throws a shadow on the claims of “specificity” of the respective compounds. A list of these studies is given in Table 3.

### 3.3. In Situ Binding

A main objection to affinity chromatography as a suitable method of target identification is that the interaction of compounds and binding proteins occurs in cell-free extracts on columns and not under physiological conditions. Consequently, chemoproteomic strategies have been developed, which circumvent this inconvenience via intracellular, thus in situ or “bio-orthogonal”, interactions of compounds with binding proteins. In one approach, the compounds of interest are conjugated to a suitable linker, such as trans-cyclooctene [105] or biotin [106], and incubated with the cells of interest. After convenient time periods, the cells are lysed, and the cell lysates are incubated with magnetic beads coated with a suitable linker (tetrazin-streptavidin [105] or streptavidin [106]), followed by pull-down and identification of the binding proteins by LC-MS/MS. Another approach uses ferric gold nanoparticles coated with the compound of interest [107]. In a different strategy, compounds of interest are conjugated to fluorophores and in situ UV-cross-linked to their binding protein partners. The cell lysates are then separated via SDS-PAGE. The fluorescent bands are isolated and the proteins identified by LC-MS/MS [108]. Bio-orthogonal studies using photoactive derivatives have identified binding proteins of various antimalarials, such as albitiazolium [109], artemisinin [110], diaminoquinazoline [111], or, more recently, probes for tagging plasmepsins [112].

### 3.4. Protein Stability-Based Methods

The methodologies described above have the disadvantage that modified compounds are used to identify binding proteins and not the original compound of interest. Rather, proteins binding to the original compound in situ, thus under native conditions, should be identified. This is possible by taking advantage of the biophysical property of proteins in which they have a higher resistance to proteolytic digestion and to chemical or thermal denaturation when bound to ligands [113].

Resistance to proteolytic digestion is exploited by a method called “limited proteolysis small molecule mapping” (LiPSMap). Mixtures of native proteins with ligands or control compounds are exposed to a protease with broad cleavage specificity, such as, e.g., proteinase K, prior to denaturation and tryptic cleavage to generate peptides for LC-MS/MS analysis. Ideally, protein domains binding to the ligands of interest but not in the controls are protected from the first digestion, and the corresponding peptides are identified [114]. This method can not only be used to identify proteins binding to endogenous metabolites but also to xenobiotics such as drugs, as exemplified elsewhere [115].

Moreover, proteins with ligands tend to denature and thus precipitate at higher temperatures than the same proteins without ligands, as illustrated by interaction with a simple fluorescent probe binding to hydrophobic residues of proteins [116]. This thermal proteome profiling (TPP) is performed using various experimental setups. In cellular thermal shift assays (CETSAs) [117], cells are incubated with the compounds to be tested, then lysed, and then the lysates are incubated for a given time period in a thermal cycler at various temperatures, starting at 37 °C [118]. Precipitated proteins are removed by centrifugation, and the supernatants are subjected to suitable functional assays [118], to one- or two-dimensional gel electrophoresis [119], or to LC-MS/MS [120,121]. Classical CETSA-MS is performed by incubating cells or lysates with the compounds to be tested or with a solvent control at ten different temperatures, followed by centrifugation of the precipitates, trypsinization and labeling of the soluble proteins with commercially available TMTs [122], or alternative labeling agents [123] and LC-MS analysis of the ten combined compound and control samples [124]. Given the large number of samples to be analyzed, this protocol is, however, limited by the manpower needed for running the system and subsequent data analysis. Consequently, simplified protocols have been developed that minimize the amounts of samples and time. One of these protocols is the isothermal shift assay (iTSA), performing thermal denaturation at a single temperature preselected for the proteome of interest [125]. Another protocol is the single tube-TPP with uniform progression (STTPP-UP). Here, incremental heating of a single sample is applied, thereby saving time and material [126]. Generally, TPP is well suited for soluble proteins. After biotinylation-based enrichment of cell surface proteins, this method can, however, be applied to surface proteins such as receptors as well [127]. Resistance to chemical denaturation, e.g., by solvents, constitutes an alternative to thermal denaturation [128]. In each experimental setup, binding proteins are identified based on their significantly increased amounts in samples with ligand vs. samples without ligand under conditions that denature the protein in the absence of ligands. In the studies quoted above, TPP has been applied on mammalian cells. During the last decade, a couple of studies using TPP have been performed with a mind to target identification in protozoal parasites. These studies are summarized in Table 4.

When evaluating thermal shift-based methodologies with respect to drug target deconvolution, one must bear in mind that the thermal stability of some proteins is also affected by binding to natural ligands such as nucleic acids and is subjected to intrinsic variation, e.g., during the cell cycle [134]. Consequently, it is imaginable that proteins binding to nucleic acids, such as ribosomal proteins, for instance, may lose their natural target upon interactions with xenobiotics and, therefore, show up in the precipitated rather than the soluble fractions of TPP assays. Moreover, compounds such as transition metal ions may stabilize proteins in solution, thereby generating false-positive results (see, e.g., [131]). A summary of affinity-based methods is given in Table 5.

## 4. Analysis of Resistant Strains

### 4.1. General Considerations

A complementary strategy for determining the mode of action of antiparasitic compounds is the analysis of resistant strains. Before going into detail, we have to define what we mean by “resistance” to a given compound. The operational definition of resistance depends on the determination of drug efficacy. One way to determine drug efficacy is to expose the organism of interest to increasing concentrations of the compounds of interest and to measure the proliferation using appropriate tools such as reporter strains (see, e.g., ref. [135] for a detailed review on *T. gondii* drug screening). Based on the proliferation data, the concentration corresponding to half of the proliferation obtained in the absence of the compound (“the inhibitory concentration 50%” or IC_50_ or other ICs, e.g., IC_90_) is then calculated by appropriate algorithms [135]. These values are compared to corresponding data obtained with suitable host cell lines (often referred to as “effective concentration” or EC_50_). The higher the EC_50_/IC_50_ ratio, the more “promising” the compound seems to be, and the more effort will be spent on subsequent investigations, such as in vivo studies including this compound. These IC_50_s are, however, only one part of the story. Another parameter of similar importance is the “minimal inhibitory” concentration (MIC), i.e., the concentration at which proliferation does not occur anymore (in other words, the IC_100_). Unlike other ICs, this IC_100_ or the MIC cannot be calculated by extrapolation from growth assay data by suitable algorithms [136]. It has to be determined by visual investigation of individual in vitro cultures exposed to increasing concentrations of the compound of interest. Another, perhaps more elegant, way is to remove the drug pressure after a convenient time period and screen for regrowth of the pathogen. Obviously, in the case of intracellular parasites, long-term exposure to the compound of interest must not harm the host cells. There is no other way to determine the MIC. Resistance to a given compound can now be defined by higher IC_50_s and higher MICs in resistant as compared to susceptible strains [137]. If only the IC_50_ is increased, but not the MIC, the corresponding strains tolerate the compound or have adapted to it but are not resistant (see, e.g., [138] or [139] for examples). Moreover, to allow robust investigation of the resistant strains, including the preparation of single clones, the resistance must not be lost in the absence of drug pressure. Due to their metabolic plasticity, eukaryotic cells (including protozoal parasites) may simply escape drug pressure by switching to a dormant stage, as observed in the case of *Plasmodium sp*. exposed to artemisinin [140]. In this case, the terms “resilience” or “tolerance” are more adequate than “resistance” [141]. Another example of resilience is given by intracellular *T. gondii* or *N. caninum* exposed to calcium-dependent kinase inhibitors. Whereas the infection of cell cultures is inhibited by these compounds at sub-micromolar concentrations, treatment of infected cells, even by one magnitude higher concentrations, does not lead to parasite death but to inhibition of egress. Subsequently, the intracellular parasites generate multinucleated complexes (see, e.g., [142]) characterized by a downregulation of more than 50% of the identified unique proteins and upregulation of a few proteins typical for dormant stages [143]. This observation cannot be generalized to all apicomplexans. *C. parvum*, for instance, is killed very efficiently by calcium-dependent kinase inhibitor treatments, even when applied post-infection [104]. This illustrates that (ultra)structural investigation of organisms treated with a given compound is paramount for correctly appreciating its effects.

### 4.2. Resistance of Transgenic Strains

The most straightforward strategy to validate potential drug targets (see Figure 3) is to perform knock-out or knock-in studies using appropriate reverse genetic tools. Overexpressing genes of interest using integrative or episomal plasmids is certainly the oldest of these methods and is well established for *G. lamblia* [144], *Plasmodium* sp. [145,146], *T. gondii* [147], and related protozoans [148]. Knock-down or silencing of genes of interest is less straightforward. RNA interference by the degradation of double-stranded RNA occurs in the excavata *G. lamblia*, *Leishmania* sp., and *Trypanosoma brucei*, as well as in the apicomplexa *Plasmodium* sp. and *T. gondii* [149], but has been supplanted as a major tool by gene editing using the CRISPR/Cas9 system in *T. gondii* [150,151], where a genome-wide screening using this method has allowed for distinguishing between essential and non-essential genes [152].

If the target catabolizes the compound of interest, overexpressors are more resistant than wildtype strains. A textbook example of such “resistance markers” are enzymes inactivating antibiotics such as beta-lactamases or neomycin phosphotransferases. Encoded by resistance plasmids (R plasmids or R factors), they spread through microbial communities and are considered a major health problem [153].

If the target protein activates the compound, overexpressors are more susceptible than wildtype strains. A good example is nitroreductases from microaerophilic or anaerobic pathogens such as, e.g., *G. lamblia* [154]. The *Giardia* genome contains open reading frames (ORFs) encoding at least four multifunctional quinone reductases with the ability to transfer electrons not only to quinones but also to nitro compounds, thereby recycling NAD or NADP cofactors [95,96,155]. The nitroreductases encoded by ORF 22677 and ORF 15307 increase the susceptibility to the nitro compound nitazoxanide when overexpressed in *G. lamblia* trophozoites and to metronidazole when overexpressed in *E. coli* [96]. In the same direction goes the overexpression of gain-of-function genes. The widely used overexpression of dihydrofolate reductase (DHFR) as a resistance marker in plasmids or in CRISPR/Cas9 gene editing constructs is such an example. The antimalarial pyrimethamine inhibits the bifunctional wildtype DHFR-thymidylate synthases of *Plasmodium* sp. and other protozoans such as *T. gondii* [68]. Overexpressors of mutated enzymes are more resistant to pyrimethamine and can be selected for on pyrimethamine-containing media.

Moreover, overexpression of potential drug targets may elicit “compensatory reactions” conferring drug adaptation or resistance, as shown for several, but, of course, not all antibiotics [156]. When analyzing data obtained with transgenic strains, one should keep in mind that these strains underwent a challenging selection for resistance against drugs effective against this parasite. Therefore, it is not surprising to observe an alteration of proteome patterns that may affect metabolism when comparing transgenic strains with wildtypes. For instance, a *Giardia* line overexpressing *E. coli* glucuronidase A differs by nearly 10% of the detected unique proteins from the corresponding wildtype with a major focus on altered patterns of surface proteins, referred to as “antigenic variation” [50]. Moreover, *T. gondii* knock-out clones of the surface antigen SAG1 created by gene editing show distinct strain-specific proteome patterns besides the intended knock-down [157].

### 4.3. Differential Analysis of the Proteomes of Susceptible vs. Resistant Strains

An alternative to the analysis of engineered stains overexpressing or silencing defined target proteins is the comparison of susceptible strains to strains resistant to a compound of interest. This strategy does not hold only for resistant pathogens but also for the analysis of chemotherapy-resistant cancer cell lines [158]. This type of study can be performed on clinical isolates [159] of a given pathogen or on resistant laboratory strains created by increasing drug concentrations in the culture medium or by chemical mutagenesis. Clearly, the analysis of clinical isolates corresponds more to “reality”, but the creation of resistant strains under laboratory conditions offers the following advantages: (i) Resistant clinical isolates can only be obtained for established drugs, not for experimental compounds. (ii) Resistance can be induced using a reference strain with well-established molecular genetic tools. (iii) The process of resistance formation can be monitored. (iv) The corresponding wildtype strain can be maintained and cultivated under the same standardized conditions as the resistant strains (except for the absence of drug in the culture medium).

The fundamental paradigm of this strategy is the investigation of *Plasmodium* strains resistant to antimalarials widely applied for prophylaxis and treatment. Fostered by genome sequencing efforts more than two decades ago [160], genomic and transcriptomic investigations have led to the identification of several gene products associated with resistance. The take-home message from these studies is that—concerning resistance to artemisinin in particular—there is no single gene or marker associated with resistance. Rather, several genes show differences with respect to point mutations or differential expression between susceptible and resistant strains, as highlighted by seminal review articles [161,162,163,164].

This view is fostered by analyses of resistant *G. lamblia* strains induced under laboratory conditions. Prima vista, the analysis of resistant patient isolates, would be closer to reality, but depending on the genotypes, the isolates are difficult to maintain in culture. Moreover, resistance may be lost after en- and excystation [165], thereby precluding the isolation of resistant strains from cyst-containing feces. Resistance to nitro drugs such as metronidazole and nitazoxanide can easily be induced by increasing drug concentrations in trophozoite culture medium, followed by cloning and characterization of the resistant clones [166,167]. Our knowledge about the anaerobic metabolism of *Giardia* and other anaerobic organisms suggests three strategies for nitro drug resistance, namely, restriction of electrons available for nitro reduction by downregulation of glycolysis, by downregulation of enzymes reducing nitro compounds (nitroreductases), or by upregulation of enzymes scavenging radicals issuing from nitro reduction [154,168]. When comparing the proteome patterns of nitro drug-resistant clones from different origins, it is striking that all three strategies are found, depending on the resistant strains and the nitro compounds used for selection [169]. The results only partially match previously published results on differential transcriptomics [170]. A summary of selected studies illustrating this strategy is given in Table 6.

As in the case of the target deconvolution studies, the overview of studies of differentially expressed proteins in resistant vs. susceptible strains presented in Table 6 reveals a large number of differentials of candidate proteins involved in the mode of action of the respective drugs.

## 5. Combining Evidence from Chemoproteomics and Whole-Cell Proteomics

So far, research designs for drug target identification that combine proteomic, in silico, and genetic approaches are gaining popularity. Is there an added value in merging outcomes of more than one proteomic approach? In other words, is it possible to combine the two strategies discussed in this review, namely, target deconvolution studies and whole-cell proteomes of resistant or resilient strains by mass spectrometry? A combination of both approaches within one model system could enhance belief in a mode of action or in the targets identified. Such studies are, however, rare. One approach is to compare the affinoproteomes of drug-susceptible and -resistant strains by a convenient method such as TPP. A first step is the analysis of thermal shifts of a favored target in susceptible and resistant strains, as exemplified by *T. gondii* susceptible or resistant to bumped kinase inhibitors and their kinase target [174]. This approach is, however, confirmative rather than explorative since the kinase target—shown by previous kinetic studies—is confirmed by another method, and other, more systemic effects of the compound are neglected. The study comparing the affinoproteomes of *L. infantum* susceptible or resistant to various antileishmanials [131] is an example of an explorative study. As mentioned in Table 4, the analysis of the affinoproteomes by TPP has revealed differentials in a good number of various proteins previously not suspected as potential targets. What would the output of a study comparing drug-binding proteins and differentially expressed proteins in the same model system look like? If drug metabolizers are the only binding proteins, one would expect to find them amongst the differentials as well. However, if components of the cellular machinery essential for survival and therefore tolerating only minor changes in expression are the main binding proteins, one would not expect them amongst the differentials. Rather, drug metabolizers or transporters [175] would be expected here.

## 6. Conclusions

Nowadays, there is a steadily increasing number of scientific articles claiming drug targets merely based on computer simulations of interactions between a single target and a single molecule. The articles reviewed above show that the identification of drug targets and, therefore, the development of reason-based chemotherapies is more complicated. It is not surprising to find multiple proteins as drug interaction partners in protozoan parasites, suggesting that the drug kills the parasite by “…disrupting its biological landscape…” as stated in a review on artemisinin-binding proteins in *P. falciparum* [176]. Indeed, the examples of studies mentioned above suggest that multiple targets interact with antiprotozoal compounds, not only the usual suspects such as specific enzymes investigated via functional assays but also components of general cellular maintenance such as cytoskeletal and ribosomal proteins or proteins involved in replication and transcription. This suggests that multigenic adaptation or resistance mechanisms get going upon exposure to these compounds and may even be part of developmental programs established during evolution as countermeasures to exposure to xenobiotics affecting fitness. The formation of multinucleated complexes in intracellular apicomplexans upon exposure to bumped kinase inhibitors may follow such a program [142].

During evolution, protozoan pathogens have learned to cope with hostile environments generated by innate or acquired host immunity, as reviewed elsewhere [177]. The onset of antigenic variation in *Giardia* [178] as a response to both drug [169] and immune [179] pressure is a good example and is worth being further exploited as a model system.

Although chemoproteomics using affinity chromatography has the potential to display a large set of drug-binding proteins, the question of how to distinguish “true” targets from drug-binding proteins remains. Certainly, experimental validation based on knockdown, knock-out, or overexpression of the target gene is feasible. Now, taking into consideration, on the one hand, the off-target molecular effects (see, e.g., [157]) and, on the other hand, the untrustworthy concept of the one drug-for one target-for one disease approach, the experimental validation of drug targets using genetic tools may ultimately leave more questions than answers.

Consequently, state-of-the-art proteomics plays an essential but not unique role in unraveling these mechanisms. More complex approaches involving, e.g., ultrastructural and metabolomic investigations of drug-treated vs. untreated and of resistant vs. susceptible strains are important to draw a more complete picture. Metabolomic investigations may even be performed on intact cells by ^1^H-NMR, as shown for *Giardia*, which again reveals its suitability as a model system to study resistance formation in protozoans [180]. This brings us to the final statement that the key to progress in studying drug-target interactions and resistance formation in protozoans, in particular, and in any empirical science, in general, is the choice of standardized model systems, well-suited for the application of state-of-the-art laboratory scale methodology.

## Figures and Tables

**Table 1:** Overview of studies presenting in vitro assays with selected target proteins.

Protein Target for Inhibitor Screenings	Pathogen	Methodology	Reference
Protein biosynthesis	*P. falciparum*	Luciferase assay	[65]
Calcium-dependent protein kinase 1	*T. gondii*	Kinase assay Cocrystallization	[66,67]
Dihydrofolate reductase thymidylate synthase	*T. gondii*	Functional assay	[68]
Dihydrofolate reductase	*P. vivax*	Heterologous expression in yeast Growth assay	[69]
Acetyl-CoA carboxylase	*T. gondii*	Functional assay	[70]
Glyoxalase 1	*T. gondii*	Functional assay	[71]
Type-II NADH dehydrogenase	*T. gondii*	Functional assay	[72]
Nucleoside triphosphate hydrolase	*N. caninum*, *T. gondii*	Chemoluminescence assay	[73]
Mitochondrial ADP/ATP	*P. falciparum*	Heterologous expression in *E. coli* Radioactive uptake assay	[74]
Importin alpha binding to nuclear localization signal	*P. falciparum*	Alpha screen binding assay	[75]
Phenylalanyl t-RNA synthetase	*T. gondii*	Functional assay	[76]
Aspartate transcarbamoylase	*P. falciparum*	Functional assay, protein interference assay	[77,78]

**Table 2:** Overview of affinity chromatography studies identifying antimalarial binding proteins. AC, affinity chromatography; BP, binding protein; DAC, differential affinity chromatography; FA, functional assay.

Antimalarial	Methodology	Remarks	Reference
Kinase inhibitors	Cell-free extracts from various cell types and organisms. DAC with active and inactive purines. SDS-PAGE followed by digestion of binding proteins and microsequencing of the peptides.	Detection of known kinases by Western blotting. Some of the peptide sequences match to other kinases and other proteins.	[91]
Quinolines	Cell-free extracts of infected human erythrocytes. DAC with ATP as a control, elution with various quinoline antimalarials, SDS-PAGE followed by Edman mixed peptide sequencing.	Human aldehyde dehydrogenase 1 and quinone reductase 2 major BP. Validated as potential target by FA.	[92]
Endoperoxides	*P. falciparum* trophozoite lysates. AC with an artemisinin analog, followed by 2-D SDS-PAGE and MALDI-TOF MS.	Identification of 9 *P. falciparum* BPs. Major BP is a calcium-binding protein.	[93]

**Table 3:** Overview of affinity chromatography (AC) and differential affinity chromatography (DAC) studies performed in protozoal pathogens and host cells. All studies were performed using epoxy-activated sepharose. BP, binding protein; FA, functional assay; GST, glutathione-S-transferase.

Organism	Ligand	Methodology	Remarks	Reference
*G. lamblia*	Thiazolide	AC, elution with ligand, SDS-PAGE followed by LC-MS/MS.	Nitroreductase NR1 major BP. Validated as a potential target by FA and in subsequent studies.	[94,95,96]
*H. sapiens* Caco2	Thiazolide	AC, elution with ligand, SDS-PAGE followed by LC-MS/MS.	Human GSTP1 major BP. Validated as a potential target by FA and in subsequent studies.	[97,98]
*H. sapiens* Fibroblasts	Thiazolide	AC, elution with ligand, SDS-PAGE followed by LC-MS/MS.	Human quinone reductase 1 major BP in *N. caninum* infected cells. Validation by FA.	[99]
*T. gondii*	Ruthenium complex	DAC with mock column only; elution by pH shift; SDS-PAGE followed by LC-MS/MS.	Translation elongation factor 1 alpha and two ribosomal proteins identified as binding proteins.	[100]
*T. gondii* *T. brucei*	Ruthenium complex	Comparative DAC with two ineffective complexes in two pathogens, elution with pH shift, LC-MS/MS on entire eluates.	128 specific *T. gondii* BPs and 46 specific *T. brucei* BPs. *Major T. brucei* BP mitochondrial ATP synthase subunit validated by FA.	[101]
*T. gondii* *M. musculus* splenocytes	Antimicrobial peptide	Comparative DAC with ineffective peptide, elution with pH shift, LC-MS/MS on entire eluates.	Several hundred BPs in eluates from both organisms, suggesting common modes of action.	[102]
*N. caninum* *D. rerio*	Bumped kinase inhibitor with quinoline core	Comparative DAC with quinine, elution with pH shift, LC-MS/MS on entire eluates.	12 specific *N. caninum* BPs and 13 specific *D. rerio* BPs. Many BPs in both organisms in quinine eluates, as well. Majority involved in RNA binding or modification.	[103]
*C. parvum* *H. sapiens* HCT-8 cells	Bumped kinase inhibitor with quinoline core	Comparative DAC with quinine, elution with pH shift, LC-MS/MS on entire eluates.	No specific binding proteins in *C. parvum*, 25 specific BPs in host cells; 29 *C. parvum* and 224 host cell BPs also in quinine eluates. Common targets in RNA binding or modification.	[104]

**Table 4:** Overview of thermal proteome profiling-based target deconvolution studies performed in protozoal pathogens. CETSA, cellular thermal shift assay; MS, mass spectrometry.

Organism	Methodology	Remarks	Reference
*Leishmania donovani*	Classical CETSA-MS on promastigotes with an inhibitor of sterol biosynthesis.	Oxidosqualene cyclase identified as a target of this inhibitor.	[129,130]
*L. infantum*	Classical CETSA-MS on cell-free extracts of amphotericin B, antimony, or miltefosine susceptible and resistant lines incubated with the respective drugs.	Up to several hundred proteins with altered melting profiles depending on the compound. Sb tends to stabilize ribosomal proteins.	[131]
*P. falciparum*	Comparison of classical and isothermal CETSA on intraerythrocytic stages using pyrimethamine as a proof of concept.	Conceptual study. No data on novel binding proteins directly available.	[132]
*T. gondii*	Classical CETSA-MS with calcium egress inhibitor ENH1 as ligand.	82 proteins with enhanced thermal stability identified, including calcium-dependent protein kinase 1.	[133]

**Table 5:** Summary of affinity-based methods identifying drug-interacting proteomes. AC, affinity chromatography; DAC, differential affinity chromatography; TPP, thermal protein profiling.

Methodology	Advantages	Inconveniences
AC—elution with ligand	Well established. Does not need sophisticated equipment. Fast.	Modification of original ligand necessary to create column matrix. Identification of major binding proteins after PAGE, resulting in low yields and bias. Cell-free extracts.
DAC—unspecific elution	See above. LC-MS/MS if elution with compatible solvent.	Needs an ineffective control compound with similar structure. Cell-free extracts.
Affinity labeling	Interaction occurs intracellularly, therefore, under physiological conditions. Fast.	Modification of original ligand necessary to create compound for affinity labeling. Polishing of labeled proteins by PAGE, therefore, low yields and bias. Label may interfere with subsequent MS.
TPP	Flexible, since interaction of proteomes and ligands is investigated under physiological conditions or in cell-free extracts. Unmodified compounds may be used.	Time and cost intensive. Use of isobaric labels. Large data volumes need appropriate bioinformatic tools.

**Table 6:** Overview of selected studies comparing whole-cell proteomes of resistant vs. susceptible strains. DIGE, differential gel electrophoresis; LC, liquid chromatography; MS, mass spectrometry; SELDI, surface-enhanced laser desorption/ionization; TOF, time of flight.

Organism	Drug	Methodology	Remarks	Reference
*G. lamblia*	Metronidazole	Comparison of three resistant cell lines created by increasing drug concentrations plus UV irradiation with susceptible parental strains. Analysis of proteomes and post-translational modifications by broad panel of proteome analytical methods.	265, 171, and 76 differentially expressed proteins depending on the strains. High isolate-dependent variability of adaptation mechanisms.	[171]
*G. lamblia*	Nitazoxanide Metronidazole	Comparison of a strain generated by increasing nitazoxanide concentrations and two metronidazole-resistant strains from study quoted above with their corresponding wildtypes. All resistant strains were resistant to both drugs and were grown in the presence of either drug prior to analysis by shotgun LC/MS-MS.	225, 248, and 304 differentially expressed proteins in the presence of nitazoxanide, 510, 287, and 216 in the presence of metronidazole. No common markers for nitro resistance. Common pattern of antigenic variation in all metronidazole-resistant vs. susceptible strains. Strategies of coping with nitro reduction strain and drug dependence.	[169]
*P. falciparum*	Chloroquine	Comparison of two clinical isolates resistant to chloroquine with two susceptible isolates using SELDI-TOF.	Study focused on the methodology. One of the susceptible strains and both resistant strains are resistant to pyrimethamine, and one resistant strain is resistant to quinine and sulfadoxine; 10 “marker proteins” identified.	[172]
*T. gondii*	Sulfadizine	Resistant clinical isolates, susceptible reference strains. Comparison of proteomes by DIGE-MS.	31 unique differential proteins were identified.	[173]
*T. gondii*	Artemisone Artemiside	Generation of resistant strains by treating the reference strain ME49 with increasing concentrations. Whole-cell-shotgun LC/MS-MS.	215 proteins downregulated in the artemisone-resistant strain, 8 proteins in the artemiside-resistant strain.	[139]

## Data Availability

New data were not created.
